# Investigating the effects of learning activities in a mobile Python tutor for targeting multiple coding skills

**DOI:** 10.1186/s41039-018-0092-x

**Published:** 2018-12-18

**Authors:** Geela Venise Firmalo Fabic, Antonija Mitrovic, Kourosh Neshatian

**Affiliations:** Department of Computer Science and Software Engineering, University of Canterbury, Private Bag 4800, Christchurch, 8041 New Zealand

**Keywords:** Python tutor, Mobile learning, Programming skills, Code writing, Code tracing, Novice students, Advanced students

## Abstract

Mobile devices are increasingly being utilized for learning due to their unique features including portability for providing ubiquitous experiences. In this paper, we present PyKinetic, a mobile tutor we developed for Python programming, aimed to serve as a supplement to traditional courses. The overarching goal of our work is to design coding activities that maximize learning. As we work towards our goal, we first focus on the learning effectiveness of the activities within PyKinetic, rather than evaluating the effectiveness of PyKinetic as a supplement resource for an introductory programming course. The version of PyKinetic (PyKinetic_DbgOut) used in the study contains five types of learning activities aimed at supporting debugging, code-tracing, and code writing skills. We evaluated PyKinetic in a controlled lab study with quantitative and qualitative results to address the following research questions: (R1) Is the combination of coding activities effective for learning programming? (R2) How do the activities affect the skills of students with lower prior knowledge (novices) compared to those who had higher prior knowledge (advanced)? (R3) How can we improve the usability of PyKinetic? Results revealed that PyKinetic_DbgOut was more beneficial for advanced students. Furthermore, we found how coding skills are interrelated differently for novices compared to advanced learners. Lastly, we acquired sufficient feedback from the participants to improve the tutor.

## Introduction

Mobile handheld devices provide distinctive features which are increasingly being utilized for learning. Pea and Maldonado (2006) summarized the unique attributes of mobile devices for learning into seven features: size and portability, small screen size, computing power and modular platform, communication ability, wide range of applications, synchronization and back-up abilities, and stylus-driven interface. These attributes are largely still relevant at this day and age, but nowadays most handheld devices, like smartphones, provide touchscreen surfaces where users can interact with directly using their bare hands without using a stylus or a thumb-pad keyboard. Smartphones are used to access course material, listen to podcasts, watch instructional videos, and communicate with peers (Dukic et al. 2015). Smartphones are also being used in classrooms, to increase interaction between the teacher and students or between students (Au et al. 2015; Anshari et al. 2017).

Python is widely used as the first programming language in introductory programming courses (Guo 2013). It is also the number one programming language in 2018 based on IEEE Spectrum Ranking (Cass and Parthasaradhi 2018). As the popularity of Python and smartphones are increasing, we aim to provide opportunities for learners to continue enhancing their Python programming skills even when they are away from personal computers. Being a mobile tutor, we hope that it would appeal to the new generation of students; allowing them to continue learning outside of a classroom setting. We present PyKinetic (Fabic et al. 2016a; 2016b), a mobile Python tutor, developed using Android SDK to teach Python 3.x programming. PyKinetic is not meant to be a stand-alone learning resource; instead, the tutor is a complement to traditional lecture and lab-based introductory programming courses. Traditional code writing exercises may be difficult on a small-screened device such as a smartphone, as the keyboard usually obstructs half of the smartphone screen. For that reason, learning activities in PyKinetic require only tap and long-click interactions.

The overall aim of our project is to design activities that will maximize learning (Fabic et al. 2017a) on a mobile device, to provide an avenue for students to continue learning even when outside of a classroom setting and/or away from a personal computer. In this paper, we present a set of learning activities that focus on debugging, code-tracing, and code-writing skills. We present a version of PyKinetic—PyKinetic_DbgOut, which contains debugging and output prediction exercises, designed to support acquisition of debugging and code tracing skills (Fabic et al. 2017b). We conducted a study with PyKinetic_DbgOut to answer the following research questions:

- (R1) Is the combination of coding activities effective for learning programming?
- (R2) How do the activities affect the skills of students with lower prior knowledge (novices) compared to those with higher prior knowledge (advanced)?
- (R3) How can we improve the usability of PyKinetic?

We will answer our research questions by providing both quantitative and qualitative evidence. The paper is structured as follows. In the next section, we present related work on mobile learning, followed by a section on coding skills. Afterwards, we describe learning activities in PyKinetic_DbgOut, followed by our experimental setup; then the findings. Following the section on findings, we present our discussion, and finally our conclusions.

## Mobile learning

Mobile learning is defined as learning that transpires in an undetermined setting or learning through mobile technologies (O'Malley et al. 2005). Park (2011) devised a pedagogical framework for mobile learning activities and classified them into four types: (1) high transactional distance and socialized (HS), (2) high transactional distance and individualized (HI), (3) low transactional distance and socialized (LS), and 4) low transactional distance and individualized (LI).

The first type (HS) is when the pedagogical strategies are mostly driven by mobile learning applications and when students are heavily participating with peers by communicating to learn together. The second type (HI) is similar to HS; the learning is directed by the mobile application, but students in this type learn individually instead of collaborating with peers. The third type (LS) is where a mobile application provides a lenient structure for learning, but students work together with their peers and teachers. Lastly, LI also provides a lenient structure like LS but students in this type learn independently. Our future goal for PyKinetic is aimed at type LI: mobile learning activities where students can independently learn Python anytime and anywhere. However, the version of PyKinetic presented in this paper can be classified as HI, as it poses a higher transactional distance (i.e., low level of learner autonomy), with a fixed order of learning activities for evaluation purposes.

Apart from one-on-one teaching like in PyKinetic, mobile learning systems are also being utilized to support diverse learning situations (Oyelere et al. 2018). Mobile applications are implemented to support an assortment of pedagogical strategies such as inquiry learning (Shih et al. 2010; Jones et al. 2013; Nouri et al. 2014; Sun and Looi 2017), flipped classrooms (Wang 2016; Grandl et al. 2018), game-based learning (Klopfer et al. 2012; Perry and Klopfer 2014; Su and Cheng 2015; Vinay et al. 2013), cooperative learning (Roschelle et al. 2010), collaborative learning (Wong et al. 2017), competition-based learning (Hwang and Chang 2016), blended classroom learning (Wang et al. 2009), exploratory learning (Liu et al. 2012), and context-aware learning (Sun e al. 2015). There are also mobile applications developed as a learning management system (LMS) (Wen and Zhang 2015; Oyelere et al. 2018), and as a massive open online course (MOOC) (Grandl et al. 2018). Moreover, the number of applications that support learning is growing rapidly in various instructional domains, such as in medicine (Vinay and Vishal 2013; Gavali et al. 2017), science (Liu et al. 2012; Klopfer et al. 2012; Jones et al. 2013; Nouri et al. 2014; Perry and Klopfer 2014; Su and Cheng 2015; Sun and Looi 2017), social science (Shih et al. 2010; Hwang and Chang 2016), language learning (Wang et al. 2009; Kim and Kwon 2012; Nakaya and Murota 2013; Sun et al. 2015; Wang 2016; Wong et al. 2017), mathematics (Roschelle et al. 2010; Wen and Zhang 2015), and computing education (Hürst et al. 2007; Karavirta et al. 2012; Boticki et al. 2013; Vinay et al. 2013; Wen and Zhang 2015; Mbogo et al. 2016; Grandl et al. 2018; Oyelere et al. 2018). Furthermore, mobile learning is proved to be effective for learning and motivating learners from various age groups, such as children and pre-teens aged between 7 and 14 (Roschelle et al. 2010; Shih et al. 2010; Liu et al. 2012; Klopfer et al. 2012; Vinay et al. 2013; Nouri et al. 2014; Su and Cheng 2015; Hwang and Chang 2016; Wong et al. 2017; Sun and Looi 2017; Grandl et al. 2018), teenage students ages 14–16 (Perry and Klopfer 2014; Wang 2016), undergraduate and graduate students aged 16–35 (Roschelle et al. 2010; Boticki et al. 2013; Nakaya and Murota 2013; Mbogo et al. 2016; Sun et al. 2015; Oyelere et al. 2018), university students and teachers (Wen and Zhang 2015), and working professionals (Wang et al. 2009).

There are many mobile applications that support various aspects of computing education such as supporting entire courses through an LMS (Oyelere et al. 2018; Wen and Zhang 2015), learning algorithm executions through visualizations (Boticki et al. 2013; Hürst et al. 2007), control-flow learning (Karavirta et al. 2012; Vinay et al. 2013; Fabic et al. 2017c; Grandl et al. 2018), and code writing (Mbogo et al. 2016). Oyelere et al. (2018) implemented MobileEdu, an LMS for third year students in a systems analysis and design course. The goal of MobileEdu was to alleviate poor engagement of students and to provide support for lecturers in managing the students. Oyelere and colleagues conducted a study where they compared students’ learning with traditional lectures (control) to students learning almost entirely through MobileEdu (experimental). MobileEdu had communication features which allowed students in the experimental group to interact with their instructor despite not having face to face lectures. Results revealed that students who learned via MobileEdu learned significantly more than the control group. They also conducted a survey which revealed that the experimental group had significantly improved perception towards the course compared to the control group. Wen and Zhang (2015) also presented an LMS referred to as Micro-Lecture Mobile Learning System (MMLS). The system contained 12 courses. The results showed that participants who used the MMLS in Data Mining, Digital Signal Processing, and MATLAB courses showed significantly higher final exam marks than previous course intakes before introducing the MMLS.

Boticki et al. (2013) also conducted a semester-long study. The authors introduced SortKo, an Android application for teaching sorting algorithms with the help of visualizations. The authors found that SortKo learners learned 30% more than those who did not use SortKo, and their survey results verified that it helped motivate the students. Hürst et al. (2007) also introduced animations to help with understanding executions of algorithms. However, the study by Hürst and colleagues was more concerned on HCI implications. They compared differences in learning between devices (laptop vs. iPod with video capability) and modalities (audio vs. no audio).

There are also several implementations in control-flow learning with different approaches. For example, Vinay et al. (2013) and Grandl et al. (2018) both had gamification approaches. However, Grandl and colleagues had a flipped classroom design where learners create various games via code like Scratch. Moreover, other implementations used Parsons problems (Parsons and Haden 2006) where syntactically correct code is given but needs to be reordered in the right order to match the given expected output (Karavirta et al. 2012; Fabic et al. 2017c). The study by Karavirta et al. (2012) was focused on supporting automated feedback for Parsons problems within their system MobileParsons, whereas our previous work (2017c) explored the effectiveness of *menu*-*based self*-*explanation* (*SE*) *prompts*. Menu-based SE prompts are learning activities which provide choices from a menu, designed to promote deeper learning by helping induce mental justifications which are not directly presented by the material (Wylie and Chi 2014).

Lastly, Mbogo et al. (2016) conducted a 2-hr-long qualitative study where their mobile learning system provided code writing exercises in Java by designing static scaffolding. The learners were able to collapse parts of the code and toggle between collapsed and full versions of their programs to support Java code writing on a smartphone. Participants remarked that the scaffolding for code segmentations made the application easier to use.

There are very few mobile learning tutors presented in the literature specifically for enhancing programming skills. Our work presented in this paper is similar to work by Karavirta et al. (2012) and Mbogo et al. (2016), with the notion of providing programming exercises to support learning. However, instead of only providing one type of activity as in those two projects, we provide various tasks to target several coding skills. Our pedagogical strategy is an implementation with a component-skills perspective (McArthur et al. 1988). We aim to fill the gap and provide an opportunity for learners to continue practicing their programming skills even when not in a lecture setting. Furthermore, we aim to provide learners an avenue to enhance several coding skills while they are away from a personal computer by supplying a combination of various coding activities.

## Coding skills

Novice programmers are slow in solving problems due to the lack of declarative and/or procedural knowledge (Anderson, 1982). In programming, declarative knowledge comprises of the syntax of the programming language and familiarity with code constructs. According to Winslow (1996), it takes 10 years for a learner to become an expert programmer. Learners often lack mental models and are unable to translate a problem into manageable tasks (Winslow 1996). The difficulty in structuring code might be evidence of a deficiency of procedural knowledge. Some students perceive code as series of instructions that are expected to execute in the specified order (Ahmadzadeh et al. 2005). Students have difficulties in comprehending the execution order, predicting the output, debugging and code writing (Pea 1986).

A variety of skills necessary for programming has been discussed in the literature. Researchers found that code tracing must be learned before code writing (Lopez et al. 2008; Thompson et al. 2008; Harrington and Cheng 2018). Further evidence proves relationships between code tracing, code writing, and code explaining (Lister et al. 2009; Venables et al. 2009). A strong positive correlation was found between code tracing and code writing (Lister et al. 2010). Harrington and Cheng (2018) conducted a study with 384 students, to investigate whether a gap exists between the ability of students to trace and write code. The study was conducted in an examination setting, where students were given two questions. The participants were randomly assigned to perform code tracing on one question and code writing on other. The results show that 56% of the students had almost no gap between their code tracing and code writing skills. For the remaining students who had of at least two out of eight marks, a strong negative correlation was found between the learners’ performance in the course and the size of the gap. Regardless of whether the student was better in either code tracing or code writing, authors suggest that a large gap was more likely due to the student struggling in the course. The authors found that underachieving students were struggling with understanding the core programming concepts. The conclusion of the authors is also supported by literature, as learners need both declarative knowledge and procedural knowledge (Anderson, 1982). Students often struggle to translate problems into manageable tasks due to the absence of mental models (Winslow 1996). Ahmadzadeh et al. (2005) conducted a study on the debugging patterns of novices and found that most learners competent in debugging were also advanced programmers (66%). However, only 39% of advanced programmers were also competent in debugging. These findings provide evidence that debugging someone else’s program requires a higher order of skill than code writing.

## Learning activities in PyKinetic

The problems in PyKinetic_DbgOut consist of the problem description, code (containing 0–3 incorrect Lines of Code LOCs), 1–3 activities, and 1–3 questions for each activity. There are five types of activites (Table 1): three types of debugging activities, and two types of output prediction activities. We first define some terminology used in the rest of the paper.

- An *activity* is an exercise based on a description, a code snippet, and possibly additional information.
- A *problem* is a task which may contain one or more coding activities, where activities are presented one at a time.
- A *test case* is an executable code which may contain more than one line of code; consists of parameters and calls to one or more functions.
- *Actual output* is the true displayed result of a program when executed, regardless of whether the code contains any errors.
- *Expected output* is the anticipated displayed result when a program which contains errors is “fixed” based on the problem requirements.

**Table 1:** Five types of debugging and output prediction activities in PyKinetic_DbgOut

Type of question	Task	Additional information given
Dbg_Read	Is the code correct? (*Yes* or *No)*	Test cases with actual output
Dbg_Ident	Identify *n* erroneous LOCs (*n* is given)	Test cases with actual output
Dbg_Fix	Fix erroneous LOCs (by tapping through given choices)	Test cases with expected output
Out_Act	Select actual output of the code	Test cases
Out_Exp	Select expected output of the code	Test cases

**Fig. 1:**
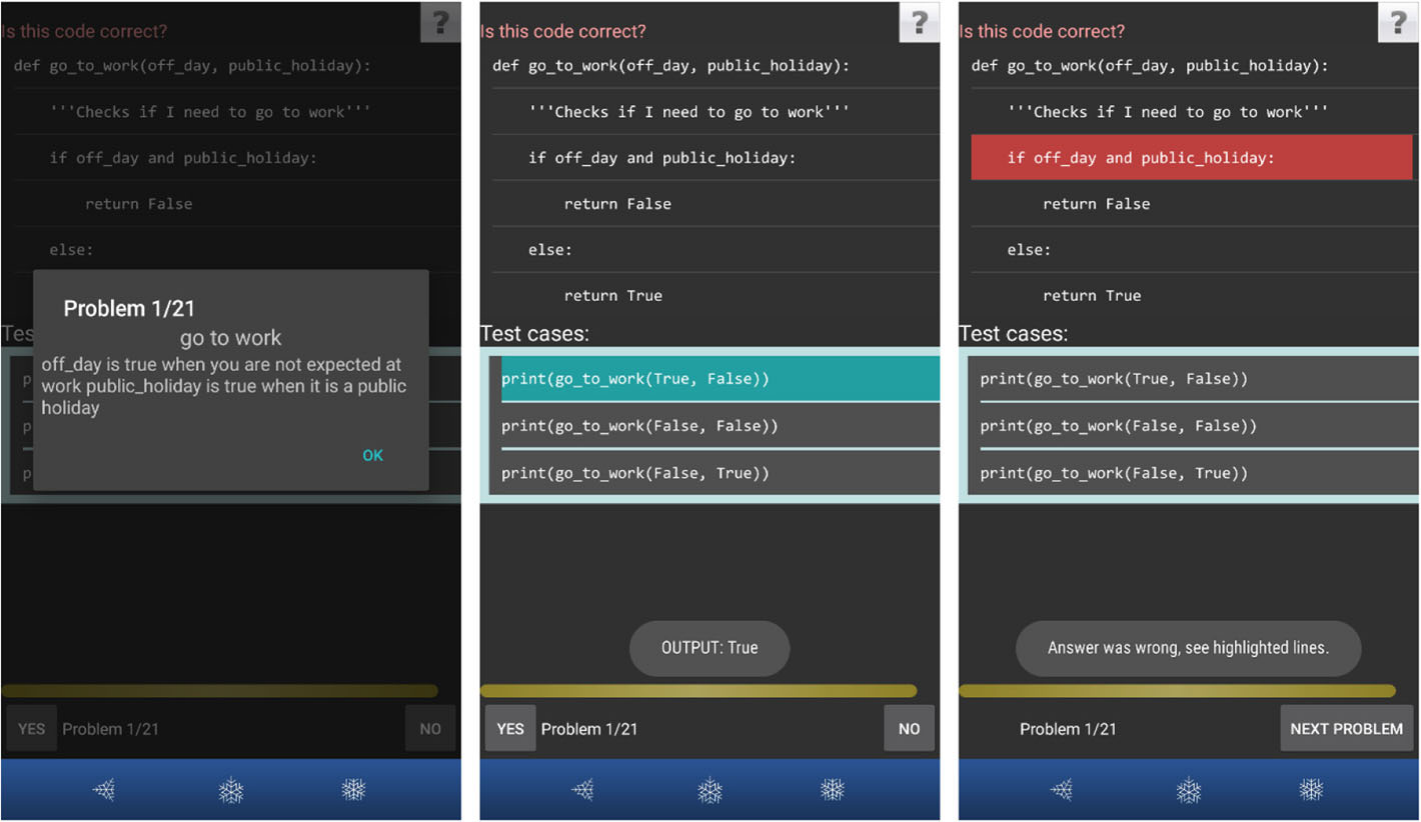
Example of a Dbg_Read activity in PyKinetic_DbgOut (left: problem description; middle: code with test cases, output shown is the actual output of the highlighted line; right: feedback when learner answered incorrectly)

In *Dbg_Read* activities, the learner is given test cases with the actual output; the learner’s task is to specify whether the given code is correct or not. Figure 1 shows an example of Dbg_Read where a learner is first given the problem description (Fig. 1, left). Function *go_to_work* should display True or False if a person is required to go to work, based on the value of two boolean parameters: *off_day* and *public_holiday*. Variable off_day is True if today is the person’s day off work, and False if it is a normal working day. Variable public_holiday is True if today is a public holiday, and False if it is regular working day. The learner then needs to respond on whether the code is error-free or not, based on the given code and the problem description. If the code contains error/s, it is not mandatory to determine the part of the code which causes the error. The learner is also given test cases where PyKinetic_DbgOut displays the actual output of a test case when executed when the learner long-presses on the test case. In Fig. 1 (middle), it shows that for the first test case, the output is True when the parameter off_day is True and public_holiday is False. The same output of True is also returned when off_day is False and public_holiday is True. The test cases show that the function contains an error since the function should only return True if the person needs to go to work. In Fig. 1 (right), the learner answered incorrectly, so PyKinetic_DbgOut highlighted the LOC causing the error in red.

**Fig. 2:**
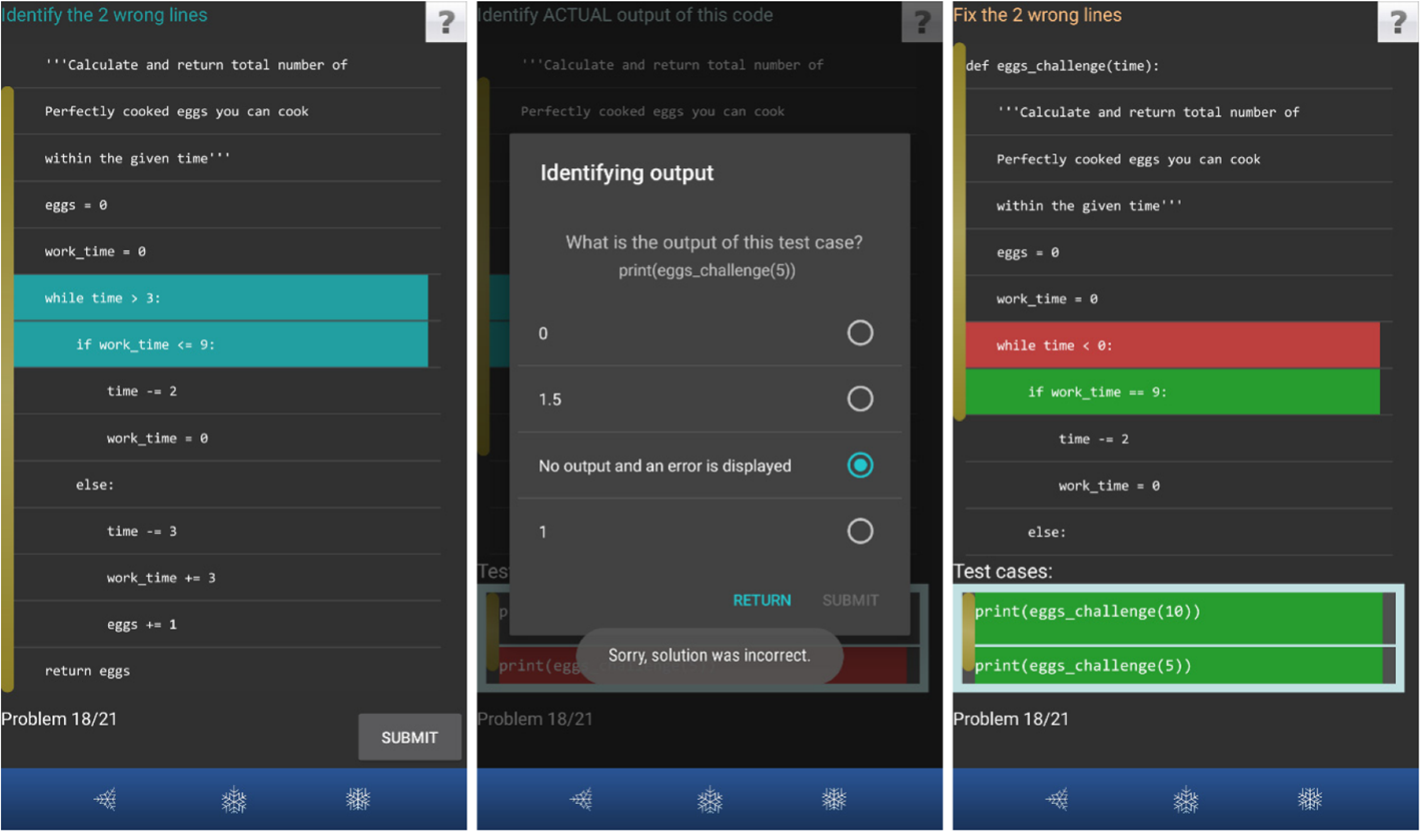
Example of Dbg_Ident –> Out_Act –> Dbg_Fix activities in PyKinetic_DbgOut (left: Dbg_Ident; middle: Out_Act; right: Dbg_Fix)

The second type of debugging activities (*Dbg_Ident*) provides similar information to the learner but instead of asking whether the code is error-free or not, the learner is made aware that the code contains errors by specifying the number of incorrect LOCs to be identified. The learner is required to identify one to three incorrect LOCs in one problem. An example is shown in Fig. 2 (left screenshot), where the student needs to identify two incorrect lines (the lines the student selected are highlighted in blue). The third type of debugging activities is *Dbg_Fix*, which starts with requiring the student to identify incorrect lines (Dbg_Ident), and then to fix them (Fig. 2, right). To fix incorrect LOCs, the student needs to select the correct option from given choices. In the screenshot shown in Fig. 2 (right), the student has completed the line highlighted in green, and needs to work on the other line (highlighted in red) as it was answered incorrectly.

Each output prediction activity contains 1–3 test cases. In the first type (*Out_Act*), the student needs to specify the actual output of the given code for each given test case (Fig. 2, middle). For example, if the code is erroneous, the actual output may be none with an error displayed (Fig. 2, middle, last option). On the contrary, in *Out_Exp* activities, the student specifies the expected code output matching the problem description. All given Out_Exp problems, first starts with a Dbg_Ident activity where the student first needs to identify the error in code.

**Fig. 3:**
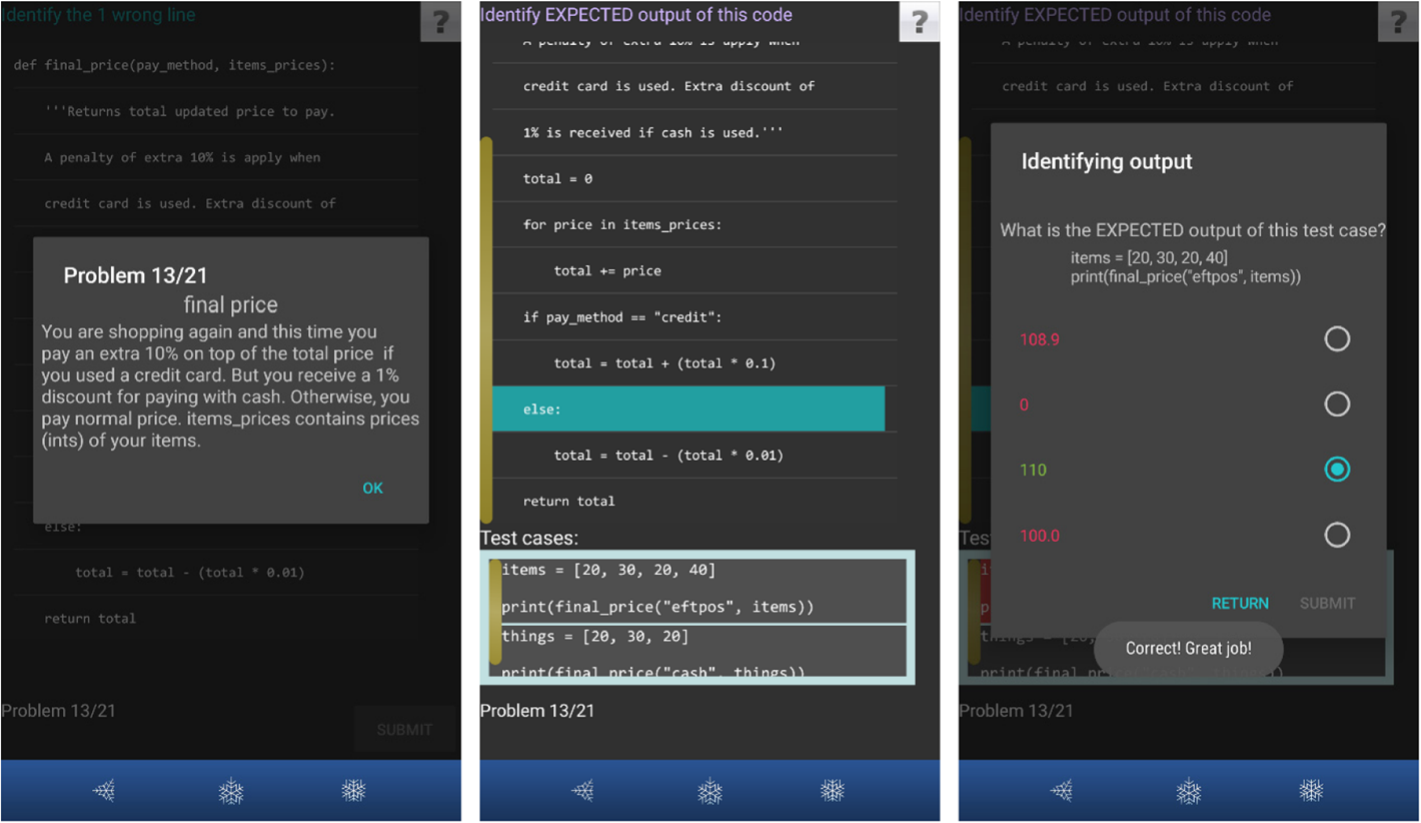
Example of an Out_Exp activity in PyKinetic_DbgOut (left: problem description; middle: code with erroneous LOC already identified, right: output prediction question for the first testcase)

Figure 3 shows an example of a problem with Out_Exp where PyKinetic_DbgOut first shows the problem description (left). The problem contains a function with two parameters: a string parameter *pay*_*method*, and a list parameter *items*_*prices*. The problem requires to sum all the values in the given list items_prices and to put the sum into the variable *total*. Afterwards, the value of total is adjusted based on the string parameter pay_method. An additional 10% is added to variable total if the variable pay_method contains the string “credit”. However, if pay_method contains the string “cash,” the variable total should be deducted by 1%. In all other cases, the value of the variable total should remain the same. Lastly, the code should return the value of total. In Fig. 3 (middle), the learner successfully identified the erroneous LOC in the code (highlighted in turquoise) and is now asked to identify the expected output of the code for each given test case. In Fig. 3 (right), the learner correctly identified the expected output in the given test case (shown in green font). Notice that because the given first parameter (pay_method) contained “eftpos,” the expected output should be the sum of all integers in the given list without any changes. If the question asked for the actual output, because of the error in the code (highlighted in turquoise in Fig. 3, middle), the answer would have been 108.9. However, the question was asking about the expected output so the correct answer was 110.

PyKinetic_DbgOut has 21 problems provided in a fixed order. There were seven levels of complexity, each containing 2–4 problems (Table 2). Problems on levels 1–3 cover conditionals, string formatting, tuples, and lists; these problems consist of 4–8 LOCs (excluding function definition, comments, and test cases), and only one activity. For example, problem one is a code reading problem, containing only one Dbg_Read activity. The complete code, problem description, and test cases with function calls are given; the task is to identify if the code is correct or not.

**Table 2:** Combinations of activities in levels 1–7

Level	Problems	Additional information given	Topics covered	Number of LOCs
1	Dbg_Read (*2 problems*)	Test cases with actual output	Conditionals	4–6
2	Dbg_Ident (*4 problems*)	Test cases with actual output	String Formatting and Conditionals	4–8
3	Out_Act (*4 problems*)	Test cases	String Formatting, Conditionals, List, Tuples	4–8
4	Dbg_Ident –> Out_Act (*2 problems*)	Test cases	String formatting, Conditionals, List, Tuples, For loops	10
5	Dbg_Ident –> Out_Exp (*2 problems*)	Test cases	String formatting, Conditionals, Lists, For loops	8–9
6	Dbg_Ident –> Dbg_Fix (*3 problems*)	Test cases with expected output	String formatting, Conditionals, Lists, For/While loops, Importing a module	9–11
7	Dbg_Ident –> Out_Act –> Dbg_Fix (*4 problems*)	Test cases	Nested While loops, Conditionals, Lists, Tuples and String Formatting	11–16

Each problem in levels 4–7 contains 2–3 activities and covered same topics as in the earlier levels as well as *for* loops, *while* loops, and importing a module. Problems on these levels started by requiring the student to identify incorrect LOCs (Dbg_Ident). After that, levels four and five are followed by output prediction activities: identifying the actual output (Out_Act) for level four and identifying the expected output (Out_Exp) for level five. Level six targets code writing skills, by requiring the student to fix erroneous LOCs (Dbg_Fix) in the second activity. The code fixing was achieved by tapping through the options with the lines changing for each tap, instead of showing a separate dialog for the options like work of Ihantola et al. (2013). Lastly, level seven contains three types of activities in each problem: identifying erroneous LOCs (Dbg_Ident), identifying actual output (Out_Act), and fixing erroneous LOCs (Dbg_Fix). The problem illustrated in Fig. 2 belongs to level seven. It is important to note that the ordering of the problems was not solely reliant on the number of LOCs and topics involved in the problem. In some cases, the code and/or the problem itself may be more logically complex than others even though it has fewer lines and topics.

## Experimental design

We conducted a controlled lab study with PyKinetic_DbgOut, focused on investigating the effectiveness of the activities in the tutor. We did not start by conducting a semester-long classroom study as we wanted to ensure the effectiveness of the activities first before exploring the effectiveness of the tutor as a supplement to a course.

### Participants

In the controlled study, we had 37 participants recruited from an introductory programming course at the University of Canterbury. We eliminated data about two participants as they have not finished the study and present the findings from 35 participants (23 males and 12 females) in the rest of the paper. The study included a brief survey on demographics. Four participants did not disclose their ages, so the average age of the rest of the participants was 21 (sd = 6.9). There was one older participant aged 54, who was found to perform similarly with other participants.

English was the first language of 23 participants (66%). Only eight participants (23%) had previous programming knowledge. We asked students to rate themselves on their programming experience on a Likert scale from one (less experienced) to ten (more experienced), and the average rating was 3.43 (sd = 1.9). We also asked students if they have previously used a Python programming tutor which is not necessarily on a mobile device, and only five participants (14%) have used one before, while the rest have not used any.

Sixteen participants (46%) owned an Android smartphone, 16 participants (46%) owned an iOS smartphone, and one participant owned both an Android smartphone and an iOS smartphone. Interestingly, two participants said that they did not own a smartphone. For those 33 participants who owned smartphones, we asked their average daily time spent on their devices; 16 participants (49%) claimed to use their smartphones for 3–8 h, 12 learners (34%) use their smartphones for 1–3 h, 3 participants (9%) use their smartphones for less than an hour, and 2 participants (6%) use their smartphones for more than 8 h.

### Method and materials

Each student participated in a single session. The sessions were 2 hr long, with 1–9 participants per session. The participants provided informed consent, followed by an 18-min pre-test, which included questions on demographics and programming background. We then gave brief instructions on using the tutor and provided Android smartphones which were mostly running on Android 6.0 (Marshmallow) and had screen sizes of 5–5.2 in with resolutions of at least 720 × 1280 pixels. Some phones had higher screen resolutions, but this was unlikely to have had any effects since the interface elements were implemented to scale relative to the screen resolution. The Android phones given to the participants already had PyKinetic_DbgOut installed. Participants interacted with the tutor for roughly an hour. Lastly, participants were given an 18-min post-test, which included open-ended questions for comments and suggestions about the tutor. The post-test was given either when time had run out, or when a participant had finished all problems. There were two tests of comparable complexity that were alternatively given as the pre-test for half of the participants. The study was approved by the Human Ethics Committee of the University of Canterbury.

The topics covered in the study have previously been covered in the lectures of the introductory programming course. The pre/post-tests had six questions each and were administered on paper. The tests contained same types of questions from Table 1 (worth one mark each), and additionally a code-writing question (worth five marks). The maximum mark for both tests is ten marks. The participants were not accustomed to doing any programming exercises on paper, because all lab quizzes and assessment in the course are completed using computers. Therefore, the code syntax on their pre/post-test were not strictly penalized. There were no multiple-choice questions in the pre/post-tests. The code-writing question provided the problem description, test cases with expected output, function definition statement, and the docstring. The code-writing questions had an ideal solution of five LOCs (without any comments), which was the reason for a maximum of five marks on this question. The participants did not receive scores for the problems completed in the tutor.

## Findings

We present the results from the 35 participants who completed the study in Table 3. There were no significant differences on any reported pre- and post-test scores in Table 3. The problems in PyKinetic_DbgOut are of different nature to the problems the participants were used to in the course, where they were mostly asked to write code. For that reason, we investigated whether there is a difference between the participants based on their code-writing skills. Before the study, the participants were assessed in a lab test, which consisted of 20 code-writing questions. The median score on the lab test was 79%. We therefore divided the participants post-hoc into 2 groups based on the lab test median: we refer to the 16 participants who scored less than 79% as novices, and to the 19 participants who scored 79% or higher as the advanced students.

**Table 3:** Pre/post-test scores (%)

Question	Pre-test%	Post-test%
Total score	68.55 (23.28)	72.88 (24.67)
Dbg_Read	77.14 (42.6)	74.29 (44.34)
Dbg_Ident	88.57 (32.28)	80 (40.58)
Dbg_Fix	57.14 (46.8)	60 (44.64)
Out_Act	93.57 (23.75)	90.71 (26.49)
Out_Exp	89.05 (23.89)	78.1 (30.99)
Code writing	56 (40.09)	69.14 (36.17)

Table 4 presents the pre- and post-test results for novices and advanced students. We used non-parametric statistical tests to compare the results, as the data was not distributed normally. The novices and advanced students spent comparable time on solving each problem. However, advanced students outperformed novices by completing more problems (U = 35, *p* = .037), and by getting higher pre/post-test scores. Although the overall pre-test score was significantly different for the two subgroups of students, there was no significant difference on the score for the code-writing question alone. Furthermore, only advanced students improved their score on the code writing question (*W* = 75, *p* = .039). On the other hand, we did not find a significant improvement for novices on the code writing question. Lastly, it seemed that output prediction questions were unfavorable for the learning of advanced students, as the advanced students had a significantly higher score than the novices on the pre-test but no significant differences between novices and advanced students in the post-test.

**Table 4:** Novices vs. advanced students (*ns* denotes not significant)

Measure	Novices (16)	Advanced (19)	*U*, *p*
Completed problems	19.63 (1.54)	20.53 (1.17)	*U* = 35, *p* = .037
Time/problem (min)	2.67 (.80)	2.82 (.44)	ns
Pre-test (%)	58.39 (21.09)	77.11 (22)	*U* = 76, *p* = .011
Post-test (%)	57.92 (28.3)	85.48 (10.75)	*U* = 63.5, *p* = .003
Improvement	ns	ns	
Normalized gain	.09 (.53)	.30 (.46)	ns
Pre-test code writing	45 (37.59)	65.26 (40.74)	ns
Post-test code writing	46.25 (41.13)	88.42 (14.25)	*U* = 76.5, *p* = .011
Improvement code writing	ns	*W* = 75, *p* = .039	
Pre-test output questions	84.11 (19.62)	97.37 (7.88)	*U* = 84.5, *p* = .024
Post-test output questions	84.9 (17.86)	83.99 (21.17)	ns
Improvement output questions	ns	*W* = 10, *p* = .022	

We calculated the Spearman’s correlations between the pre- and post-test scores of novice students. Novices had a significant positive correlation on pre- and post-test scores on all questions (*r*_*s*_ = .61, *p* = .012). However, novices had a significant negative correlation on their pre- and post-test scores on output prediction questions (*r*_*s*_ = − .53, *p* = .036). Furthermore, we found no significant correlation between the pre- and post-test scores of novices on debugging questions. There were no significant correlations between pre- and post-test scores for advanced students.

### Performance in PyKinetic

We present a comparison of the performance measures of novices and advanced participants within PyKinetic_DbgOut in Table 5. As some problems contain multiple activities, we report the performance measures for each type of activities, not per problem. We did not calculate the performance of the participants on the code reading activity (Dbg_Read), since we only provided two of these questions, and we only allowed participants to attempt these once, as these are true or false questions. The average attempts reported on Table 5 were based on the number of submissions on each activity. We have calculated the normalized gain using two formulas. When the learning gain was positive, we calculated the quotient of gain (post-test score – pre-test score) and (100 – pre-test score). However, when the learning gain was negative, we calculated the quotient of gain and the pre-test score.

**Table 5:** Novices vs. advanced students performance measures (*ns* denotes not significant)

	Identifying error	Output prediction	Code fixing
Attempts			
Novices	5.33 (2.98)	3.26 (.51)	8.07 (4.02)
Advanced	4.21 (.94)	2.63 (.28)	5.37 (1.52)
	ns	*p* = .000, *U* = 256	*p* = .002, *U* = 243
Time per activity (min)			
Novices	2.01 (0.69)	1.48 (0.63)	1.63 (0.82)
Advanced	2.23 (0.43)	1.30 (0.30)	1.59 (0.47)
	ns	ns	ns
Time per attempt			
Novices	0.87 (0.47)	0.56 (0.27)	0.25 (0.14)
Advanced	0.99 (0.32)	0.56 (0.15)	0.38 (0.14)
	ns	ns	*p* = .003, *U* = 65
Score			
Novices (16)	0.47 (0.16)	0.63 (0.13)	0.25 (0.16)
Advanced (19)	0.60 (0.16)	0.75 (0.09)	0.47 (0.15)
	*p* = .020, *U* = 82.5	*p* = .006, *U* = 70.5	*p* = .000, *U* = 40

The novice students made significantly more attempts in comparison to advanced students on output prediction and code fixing activities, but not for identifying errors. There was no significant difference on the time spent per activity between two groups of students. The average times per attempt for novices and advanced students were similar apart from the average time on the code fixing activity. The score on each activity was calculated based on the number of correct answers in their first attempt. The maximum score for each activity is 1. For example, in an identifying error activity (Dbg_Ident), if there were three errors to be identified, all three should have been identified correctly on the first attempt to get a score of 1. If only two out of three were correct, a score of 0.67 was given, and a score of 0.33 if only one out of three was correct. However, if the activity only had one required answer, then the score given will be either 0 or 1. The advanced students outperformed the novices on the average score based on their first attempts, on all three activities in Table 5.

We also investigated whether there were any correlations between the scores on the first attempt on an activity and the post-test performance. Table 6 presents significant correlations for average scores based on the first attempt on the following activities: identifying error, output prediction, and code fixing. We also present significant correlations between the average scores and the normalized gains. All reported correlations for advanced students are moderate to high positive correlations. Results revealed that the average scores of advanced students on identifying errors strongly correlated with their scores on output prediction (*r*_*s*_ = 0.68, *p* = .001). Their scores on identifying errors also showed a moderate positive correlation with their scores on fixing code (*r*_*s*_ = 0.56, *p* = .014). On the contrary, there were no significant correlations for novices. There were no significant correlations between the average scores on output prediction and average scores on code fixing for both the advanced and novice students. Based on the results presented on Table 6, advanced students benefitted most with identifying error activities as it revealed statistically significant medium to strong positive correlations with their post-test performance on several types of questions: all questions together, code writing, and output prediction questions. Furthermore, we found a medium positive correlation between scores of advanced students on output prediction and their normalized gains on output prediction (*r*_*s*_ = 0.55, *p* = .016). This was unexpected since advanced students showed a significant decrease with their scores on pre- to post-test on output prediction questions (Table 4).

**Table 6:** Spearman’s correlations between scores, post-test and normalized gains (*ns* denotes not significant)

	Novices (16)	Advanced (19)
Correlation on *score on identifying error* and:		
Normalized gain	ns	*r*_*s*_ = 0.55, *p* = .016
Normalized gain on output prediction	ns	*r*_*s*_ = 0.58, *p* = .009
Normalized gain on code writing	ns	*r*_*s*_ = 0.46, *p* = .048
Post-test code writing	ns	*r*_*s*_ = 0.59, *p* = .008
Post-test all questions	ns	*r*_*s*_ = 0.66, *p* = .002
Post-test output prediction	ns	*r*_*s*_ = 0.55, *p* = .015
Correlation on *score on output prediction* and:		
Score on identifying error	ns	*r*_*s*_ = 0.68, *p* = .001
Normalized gain on output prediction	ns	*r*_*s*_ = 0.55, *p* = .016
Post-test output prediction	ns	*r*_*s*_ = 0.55, *p* = .015
Correlation on *score on code fixing* and:		
Score on identifying error	ns	*r*_*s*_ = 0.56, *p* = .014

**Fig. 4:**
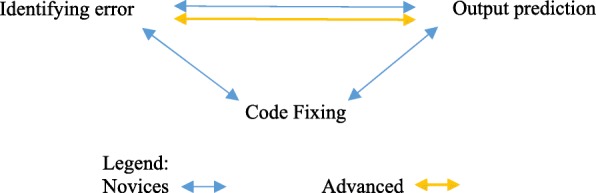
Relationship between activities for novices vs. advanced students

The results shown in Table 6 seem to show that the disparity between novices and advanced students is astoundingly evident. So, we were wondering whether advanced students were just better on their first attempts because of higher prior knowledge. Therefore, we delved deeper and considered average performance on the entire duration of each activity rather than focusing only on first attempts. More specifically, we calculated correlations between the average time per attempt on each activity type with the average normalized gains (Table 7). We found significant high positive correlations between time per attempts on identifying error, output prediction, and code fixing. However, there were clear differences between the correlations found in novices and advanced students. For novices, time per attempt on identifying error was positively correlated with their time per attempt on output prediction (*r*_*s*_ = 0.81, *p* = .000). Furthermore, their time per attempt on identifying error is also positively correlated with their time per attempt on code fixing (*r*_*s*_ = 0.70, *p* = .002). Finally, their time per attempt on code fixing is also positively correlated with their time per attempt on output prediction (*r*_*s*_ = 0.67, *p* = .004). For advanced students, we only found one significant correlation between their time per attempt within the three activities which was between identifying error and output prediction (*r*_*s*_ = 0.66, *p* = .002). Notice that the correlations between time per attempt on identifying error and time per attempt on output prediction were both significant for novices and advanced students. However, for novices, we obtained a stronger positive correlation (*r*_*s*_ = 0.81, *p* = .000) compared to advanced students (*r*_*s*_ = 0.66, *p* = .002). To sum up, all three activities are positively correlated with each other for novices, but only identifying error and time per attempt was significant for advanced students (Fig. 4).

**Table 7:** Spearman’s correlations between time per attempt and normalized gains (*ns* denotes not significant)

	Novices (16)	Advanced (19)
Correlation on *time per attempt on identifying error* and:		
Time per attempt on output prediction	*r*_*s*_ = 0.81, *p* = .000	*r*_*s*_ = 0.66, *p* = .002
Normalized gain on all questions	ns	*r*_*s*_ = 0.50, *p* = .031
Normalized gain on output prediction	ns	*r*_*s*_ = 0.55, *p* = .014
Normalized gain on code writing	ns	*r*_*s*_ = 0.61, *p* = .005
Post-test output prediction	ns	*r*_*s*_ = 0.48, *p* = .037
Correlation on *time per attempt on output prediction* and:		
Time per attempt on code fixing	*r*_*s*_ = 0.67, *p* = .004	ns
Normalized gain on output prediction	*r*_*s*_ = 0.52, *p* = .038	ns
Correlation on *time per attempt on code fixing* and:		
Time per attempt on identifying error	*r*_*s*_ = 0.70, *p* = .002	ns

A similar disparity (like reported values in Table 6) was revealed between novices and advanced students when correlations between time per attempts and normalized gains were calculated (Table 7). However, unlike the values reported in Table 6, we found one significant correlation for novices between time per attempt on output prediction and normalized gain on output prediction (*r*_*s*_ = 0.52, *p* = .038). On the other hand, for advanced students, similar significant correlations are reported on Tables 6 and 7. However, in Table 7, all significant correlations for advanced students were only on their time per attempt on identifying errors. Furthermore, most of the significant correlations were on normalized gains apart from one correlation with post-test output prediction (*r*_*s*_ = 0.48, *p* = .037).

Since we used both scores and time per attempts to calculate correlations, we also calculated correlations between these measures (Table 8). We found related results for advanced students as in Table 7. Table 8 shows significant positive correlations for advanced students, but only between activities of output prediction and identifying errors (*r*_*s*_ = 0.51, *p* = .026) as visualized in Fig. 4. Furthermore, correlations were found between scores and time per attempt of advanced students for both activities of identifying errors (*r*_*s*_ = 0.71, *p* = .001) and code fixing (*r*_*s*_ = 0.55, *p* = .015). For novices, values in Table 8 also show significant correlations between identifying errors and output prediction (*r*_*s*_ = 0.56, *p* = .025; *r*_*s*_ = 0.50, *p* = .049), as well as identifying errors and code fixing (*r*_*s*_ = 0.53, *p* = .036). Moreover, a correlation was found between score and time per attempt on output prediction (*r*_*s*_ = 0.56, *p* = .024). There were no correlations found between code fixing and output prediction for novices when looking at scores and time per attempt, unlike in Table 7 (*r*_*s*_ = 0.67, *p* = .004).

**Table 8:** Spearman’s correlations between score and time per attempt (*ns* denotes not significant)

	Novices (16)	Advanced (19)
Correlation on *score on identifying error* and:		
Time per attempt on identifying error	ns	*r*_*s*_ = 0.71, *p* = .001
Time per attempt on output prediction	*r*_*s*_ = 0.56, *p* = .025	ns
Time per attempt on code fixing	*r*_*s*_ = 0.53, *p* = .036	ns
Correlation on *score on output prediction* and:		
Time per attempt on identifying error	*r*_*s*_ = 0.50, *p* = .049	*r*_*s*_ = 0.51, *p* = .026
Time per attempt on output prediction	*r*_*s*_ = 0.56, *p* = .024	ns
Correlation on *score on code fixing* and:		
Time per attempt on code fixing	ns	*r*_*s*_ = 0.55, *p* = .015

### Analyses based on demographics

We used the Mann-Whitney *U* test to check differences between the participants based on demographics, focusing on gender, whether English was their first language, and personal smartphone device. There were 11 advanced and 12 novice male students, and there were 8 advanced and 4 novice female students. When results of male and female students were compared, no measures were significantly different.

We performed the Mann-Whitney *U* tests between participants who had English as their first language (23) to those who had another language (12). The participants with English as their first language had significantly higher scores on the following: pre-test on expected output prediction (*p* = .041, *U* = 79.5), pre-test on all output predictions (*p* = .037, *U* = 78.5), post-test scores on code writing (*p* = .049, *U* = 81.5), score on code reading in PyKinetic_DbgOut (*p* = .007, *U* = 61), score on output prediction in PyKinetic_DbgOut (*p* = .037, *U* = 78.5), and score on code fixing in PyKinetic_DbgOut (*p* = .002, *U* = 53). Note that all scores within PyKinetic_DbgOut were based on the correctness of their first attempt on each activity. Furthermore, 15 (65%) participants who had English as their first language were advanced students, whereas only (33%) 4 participants had another first language who were also advanced students.

When comparing results of participants based on their personal smartphone devices with Android (16) compared to iOS (16) users, participants who used Android devices had a significantly higher pre-test score on code fixing questions (*p* = .002, *U* = 48), and pre-test score on debugging questions (*p* = .002, *U* = 47.5). The Android users also had a significantly higher score on identifying errors within PyKinetic_DbgOut based on their first attempt (*p* = .035, *U* = 72). Contrary to that, Apple users were significantly faster on their average time per problem with an average of 2.49 min (sd = .52) and average of 2.91 min (sd = .67) for Android users (*p* = .023, *U* = 68). However, Apple users were not significantly faster than Android users on their average time per activity. Furthermore, we examined whether there was a correlation on the length of average use of participants with their smartphones with their normalized gain scores, but we did not find any significant correlations.

When comparing novices with advanced students, their estimated programming experience was statistically comparable. No significant correlations were found between indicated programming experience by participants and their normalized gains. Also, no significant correlations were found between indicated programming experience by participants and their scores within PyKinetic_DbgOut. However, we found correlations between programming experience as stated by participants and some of their pre-test scores: on code writing (*r*_*s*_ = 0.36, *p* < .05).

### Comments and suggestions from participants

The post-test included a few questions about PyKinetic_DbgOut. We asked participants an open-ended question on whether they would like to use PyKinetic_DbgOut again and to state their reasons on why they would or would not do so (Q1). Three participants did not respond to the question. Twenty-seven participants (77%) said that they would like to use it again (including 1 participant who gave a condition that he/she will only do so if PyKinetic_DbgOut was improved with more feedback), and 5 participants (14%) said that they would not. Some of the participants who refused to use PyKinetic_DbgOut again mentioned that they learn better by writing their own code. However, despite their preference to practice on a personal computer, three out of these five participants showed an improvement in their learning in using PyKinetic_DbgOut based on their scores from pre- to post-test.

The questionnaire also contained an open-ended question asking participants for any comments or suggestions for PyKinetic_DbgOut (Q2). Since we already asked the participants to state their reasoning (Q1) on wanting (or not) to use PyKinetic_DbgOut again, some participants wrote their comments and suggestions as an answer to (Q1). For this reason, we combined all the comments and suggestions from both (Q1) and (Q2) and classified them. Some participants wrote multiple comments, hence the reason for the numbers below. We classified the comments into following categories:

- (C1) PyKinetic_DbgOut was helpful and/or had a good range of content
- (C2) PyKinetic_DbgOut is good for debugging and code understanding
- (C3) PyKinetic_DbgOut is intuitive and has a good user interface
- (C4) Some questions were too difficult.
- (C5) PyKinetic_DbgOut is convenient especially when not near a computer

Category C1 contains comments from 23 participants (63%), such as *It was fun and helpful* and “There are very good questions with a good degree of challenge.”. Category C2 contains comments from six participants (17%), examples of which are “It is good for extra practice and I know how to point out mistakes and fix them after using it.” and “It was good practice at reading a program and understanding what it does.” Category C3 contains comments the usability and user interface of the tutor from four participants (11%), such as “It is quick to pick up and very intuitive”. Three participants provided negative remarks (C4), who found some of the questions in PyKinetic_DbgOut too difficult. Lastly, in category C5, there are comments from two participants (6%), who noted that PyKinetic_DbgOut is convenient to use when not near a computer. There were some interesting comments. One of the participants commented about the interface: “I felt the app made good use of the limited screen space.”

We also combined the suggestions and classified them as follows:

- (S1) Feedback needs to be improved
- (S2) Include code writing questions
- (S3) Usability can be improved particularly in code fixing
- (S4) Implement gamification on PyKinetic_DbgOut

The most common suggestion (S1), made by ten participants, was to improve feedback, such as “Give some guide notes when people made mistakes.” Seven participants suggested to add some code writing questions (S2), as some of them mentioned that the activities in PyKinetic_DbgOut does not fit their style of learning. Six participants mentioned that the usability of the PyKinetic_DbgOut could be improved particularly for the code fixing activity (S3). Some of them mentioned that they found it easy to accidentally submit a random answer by mistake when fixing LOCs. Finally, three participants suggested to add gamification (S4), specifically to add a scoring and penalty system, as it was easy to cheat via trial and error, specifically on the code fixing activity. A notable suggestion from one participant was the following: “Potential integration into a lecture environment would be very cool. ☺”. Another participant mentioned a similar suggestion: “The tutor would be good to use for testing students where small simple tests might be appropriate, such as during lectures when the lecturer may want to ask students to interact with the content.”

## Discussion

We found several differences between participants with lower prior knowledge (novices) and those with higher prior knowledge (advanced). We reported differences between their learning improvement, performance in PyKinetic_DbgOut, and relationships between their coding skills. We discuss those differences separately in the three subsections (“Learning improvement” to “Relationships between coding skills”) to follow. In the last subsection “Analysis with demographics,” we discuss findings when participants were grouped based their demographics.

### Learning improvement

Our results revealed significant correlations on the pre- and post-test scores of novices, implying that novice learners mostly relied on their prior knowledge to answer the post-test. Significant positive correlations were found on the following: pre- and post-test on all questions, and pre- and post-test on output prediction questions. However, for the advanced students, there were no significant correlations on any of the pre- to post-test scores, including pre- and post-test scores on debugging questions. Since most activities in the tutor are mostly composed of debugging activities, we suspect that the reason why only advanced students showed significant improvement in learning was because of the hierarchy of coding skills. As discussed in “Coding skills” section, researchers found that code tracing needs to be mastered before being able to be proficient in code writing (Lopez et al. 2008; Thompson et al. 2008; Harrington and Cheng 2018). Furthermore, Ahmadzadeh et al. (2005) found that debugging code written by someone else requires higher order of knowledge than code writing. Therefore, we have seen evidence of the coding hierarchy of skills in our study. We propose that this may not necessarily mean that the activities were too difficult for novices. Instead, it seems to be that learning with activities on the higher end of hierarchy of programming skills proved to be not beneficial for novices. This is because they are most likely lacking the understanding of the core concepts of programming, and unless these are learned, students may not be able to benefit. Like what Anderson (1982) mentioned, both declarative and procedural knowledge is needed by students.

We presented more evidence showing that advanced students learned more than novices with PyKinetic_DbgOut, based on the correlations between their scores in the tutor and their post-tests, and their scores in the tutor when correlated with their normalized gains. The correlations revealed that identifying error activities benefitted the advanced students most, as it positively correlated with their post-test scores and normalized gains in various activities. Similar correlations were found when we used scores as a measure, and time per attempt as presented in Tables 6 and 7 respectively. It appeared that advanced students performed worse on pre- to post-test on output prediction questions (Table 4). However, we found a significant positive correlation between their scores on output prediction in the tutor with their normalized gains on output prediction (*r*_*s*_ = 0.55, *p* = .016). We propose that the learning benefit was most likely not reflected on the post-test due to the ceiling effect on output prediction problems (pre-test avg. = 97.37%, sd = 7.88). The score of advanced students for identifying errors was also correlated with their normalized gain for all questions and their normalized gain on code writing. These results are consistent with the literature as (Ahmadzadeh et al. 2005) found that most learners with good debugging skills are also advanced programmers.

### Performance in PyKinetic_DbgOut

The differences between the performance of novices and advanced students in PyKinetic_DbgOut were interesting. Firstly, one might expect that advanced students will be faster than novices, but we found that there were no significant differences with the average time they spent in the three activities: identifying errors, fixing errors, and output prediction. It might have been because, regardless of their abilities, the participants were not accustomed to doing activities in PyKinetic_DbgOut in their course, as they were mostly doing code writing. However, the advanced students outperformed the novices when we calculated their scores based on their first attempts. Furthermore, novices had significantly more attempts on output prediction and code fixing activities. This leads us to believe that some novices might be using trial and error strategies. Furthermore, a trial and error strategy are most likely being utilized more in the code fixing activity as it was revealed that novice students had a significantly lower time per attempt than advanced students in this activity. This result suggests that they completed the code fixing activities with more attempts but with less time, in comparison with the advanced students. Furthermore, although they did not admit to using a trial and error strategy, some participants mentioned in their suggestions that we should improve the usability specifically for the code fixing activity. Participants found that the controls in the code fixing activities made it easy to use a trial and error strategy. Therefore, we should improve PyKinetic by providing support for optimal strategies, to prevent learners from guessing.

### Relationships between coding skills

We presented correlations between time per attempts and normalized gains for both the novice and advanced students. Bearing in mind that learners were not allowed to skip any activity, the average time per attempt is indicative of the performance of the learners. We showed evidence that the performance of novices on debugging code is positively correlated with their performance in tracing code. Moreover, their performance on code tracing is also positively correlated with their performance on fixing code. Lastly, their performance on fixing code is also positively correlated with their performance in debugging. All these are significant strong correlations, showing evidence that students with lower prior knowledge perform similarly across various coding activities of debugging, tracing and fixing code. However, we did not yield the same result between code tracing and code fixing when we considered both score and time per attempt (Table 8). However, we think that this might be related to the possible issue of controls in code fixing activities mentioned by the participants (“Comments and suggestions from participants” section). Therefore, we have reasons to accept that our illustration in Fig. 4 still holds. Our results showing relationships between skills is consistent with literature as Harrington and Cheng (2018) also found indications that underachieving students usually have a large gap between coding skills as they are most likely struggling in core programming concepts. Core programming concepts are essential in learning coding skills of debugging, tracing, and fixing. We see further confirmation of this when we looked at correlations between the same measures for students with higher prior knowledge. For advanced students, only their time per attempt on debugging revealed a significant correlation with code tracing. The performance of advanced students in identifying errors showed a strong positive correlation with their performance on output prediction, which demonstrated the relationship between debugging and code tracing skills (Table 7). Similarly, a moderate positive correlation was shown between debugging and code tracing when we had taken both scores and time per attempts into consideration (Table 8). Our results demonstrated how various coding skills are interrelated and how it differs between students of varying prior knowledge.

### Analysis with demographics

We also performed analysis by grouping the participants based on their demographics. We did not find any significant result when comparing male and female students, most likely because of the lower proportion of female students. When we compared participants based on whether English was their first language, it seemed like participants who had English as their first language performed better than the other students. However, it was probably due to the English group having more advanced learners compared to the other group. We also compared the participants based on their personal devices, Android users compared to iOS users. Android users achieved a significantly higher score on identifying errors within PyKinetic_DbgOut but this was most likely because Android users had a significantly higher pre-test score on debugging questions. Lastly, one might expect regardless of the smartphone that they are using, learners who often use their smartphones would learn more in using a mobile tutor such as PyKinetic_DbgOut. However, we found that was not the case. This might be an indication that PyKinetic_DbgOut can be useful to a learner despite their limited smartphone experience. We also did not find any significant correlations on their average use of their smartphones with their scores within PyKinetic_DbgOut.

## Conclusions

We presented a controlled lab study which investigated the effectiveness of activities in PyKinetic: PyKinetic_DbgOut and our findings from the study. We had three research questions for this study. Firstly, we investigated whether the combination of coding activities is effective for learning programming (R1). We found that the group learned from pre- to post-test, but the learning gain was not statistically significant. So, we did a post-hoc division of the participants by their median scores from their course lab test, as we believe this is gives a better representation of their knowledge in programming. Participants who scored less than the median were labeled as novices (16), and the rest as advanced students (19). When we repeated the analysis, we found that only advanced students showed significant learning improvement. Therefore, we found enough evidence to answer our first research question (R1) and found that the combination of coding activities was effective, but primarily for advanced students.

In our second research question (R2), we explored on how the activities affected novices and advanced students. We reported evidence that for novices, all three activities (identifying errors, output prediction, and code fixing) are interrelated with each other. We showed that these activities were positively correlated with each other, showing evidence that if for example a novice learner is proficient at identifying errors, he/she will also be skillful in output prediction, and fixing code. This can also be interpreted that debugging, code tracing, and a subset of code writing skills are interrelated for novices. We found that although their performance on the activities seem to be equally related, novices did not show any evidence that it was correlated to their learning gains (Table 6). On the other hand, advanced students showed evidence that only their performance on identifying errors and output prediction was positively correlated. Therefore, debugging and code tracing skills are interrelated for advanced students. Furthermore, we found evidence that the performance of advanced students in PyKinetic was correlated with their normalized gains. More importantly, activities on debugging (identifying errors) seemed to be most beneficial for advanced students. We presented evidence that their performance on those activities were positively correlated with their normalized gain for all questions, and for specific activities: output prediction, and code writing. Therefore, one of the contributions of the paper is that we confirmed results from the literature that there is a hierarchy in programming skills; evident even when learning via a mobile tutor. Firstly, as mentioned by Anderson (1982), learners need both declarative and procedural knowledge. Specifically, code tracing needs to be learned before writing code (Lopez et al. 2008; Thompson et al. 2008; Harrington and Cheng 2018), and debugging someone else’s program requires higher order of skill than code writing (Ahmadzadeh et al. 2005). We reported evidence which supports work of Ahmadzadeh et al. (2005). Our second contribution is that we also confirmed that debugging someone else’s programs is beneficial for advanced students. Moreover, another contribution is that we have provided evidence showing that programming can also be learned on a mobile phone, even when (in our study) they only learned with PyKinetic for an hour. We also reported sufficient evidence that the same programming hierarchy of skills applies when learning through a mobile.

We also asked the research question of how we can improve the usability of PyKinetic (R3). Firstly, we found that the daily smartphone usage of the participants was not correlated with their learning gains in PyKinetic. Furthermore, their smartphone experience was also not correlated with their scores within the app. This could be an indication that the tutor already has a user-friendly interface since we did not find any evidence that their performance and learning gains are dependent on their experience with smartphones. Moreover, we asked the participants a few questions on whether they would use it again and asked for comments and suggestions. Overall, we received a positive response from the participants. Four participants complimented PyKinetic that it was intuitive and easy to use. Furthermore, 63% (22) of the participants said that the tutor was helpful and/or had a good range of content. However, three participants said that they found some of the questions too difficult.

We also found helpful suggestions to improve PyKinetic. The most popular suggestion was that the feedback needs to be improved. We expected this, as we had time limitations for implementation which only allowed us to provide one pre-determined feedback for each activity. The second most popular suggestion from six participants was to include code writing questions. Although work was done in providing code writing exercises on a phone (Mbogo et al. 2016), the evaluation presented qualitative results without investigating learning effects. Therefore, we are not convinced that it can be effective for learning in a smartphone. More work needs to be done in this area. Furthermore, six participants also remarked that the code fixing exercise was easy to cheat, and it was easy to submit an answer by mistake. This was an unexpected suggestion and will be taken it into consideration for improvement. Enhancing the code fixing activity is relevant to the last suggestion which was to implement game features in PyKinetic. If we add game features, we can for example add limitations on the number of tries when solving a code fixing activity. The findings from this study enabled us to develop an adaptive version of PyKinetic which had personalized problem selection (Fabic et al. 2018).

PyKinetic is designed to improve coding skills in Python, and our findings support this. Our research ultimately aims to fill the gap in the literature and investigates the effectiveness of a combination of several coding tasks within a mobile tutor to provide learners with opportunities to continue enhancing their programming skills while away from computers and classrooms. The limitations of this study include the small set of participants, and limited feedback provided by PyKinetic. Our future work includes repeating the study with more participants. We also plan to conduct a longer study to investigate the effectiveness of PyKinetic as a supplement for a course. Such a study would allow participants to use PyKinetic for as much as they want, since our future goal for PyKinetic is to develop it with low transactional distance and individualized learning activities (LI) (Park, 2011). We aim for PyKinetic to be a supplement to introductory programming courses where learners can independently hone their Python skills anytime and anywhere. Furthermore, we aim to continue developing PyKinetic with a component-skills pedagogical strategy (McArthur et al., 1988) and provide various tasks which are effectively sequenced to target multiple coding skills.

## Abbreviations

LMS: Learning management system
LOC: Line of code
MMLS: Micro-Lecture Mobile Learning System
MOOC: Massive open online course
SE: Self-explanation
